# The prevalence of chronic kidney disease in South Africa - limitations of studies comparing prevalence with sub-Saharan Africa, Africa, and globally

**DOI:** 10.1186/s12882-023-03109-1

**Published:** 2023-03-21

**Authors:** Sudesh Hariparshad, Rajendra Bhimma, Louansha Nandlal, Edgar Jembere, Saraladevi Naicker, Alain Assounga

**Affiliations:** 1 Department of Nephrology, College of Health Sciences, Nelson R Mandela School of Medicine, University of KwaZulu-Natal, Durban, South Africa; 2 Department of Paediatrics and Child Health, College of Health Sciences, Mandela School of Medicine, Nelson R, University of KwaZulu-Natal, Durban, South Africa; 3 Discipline of Optics and Imaging, College of Health Sciences, Nelson R Mandela School of Medicine, University of KwaZulu-Natal, Durban, South Africa; 4 School of Mathematics, Statistics and Computer Science, University of KwaZulu-Natal, Durban, South Africa; 5 Department of Internal Medicine, School of Clinical Medicine, Faculty of Health Sciences, University of Witwatersrand, Johannesburg, South Africa

**Keywords:** Prevalence, Epidemiology, Chronic kidney disease, Renal insufiency, Renal impairment, Nephropathy, Stage III-V CKD, Proteinuria, Albuminuria, Meta-analysis, Systematic reviews, Cohort, Cross-sectional, South Africa, Sub-saharan Africa, Africa, Global

## Abstract

**Background:**

Chronic kidney disease (CKD) is a globally significant non-communicable disorder. CKD prevalence varies between countries and within a country. We compared the prevalence rates of CKD in South Africa with sub-Saharan Africa, Africa, and globally.

**Methods:**

We registered a systematic review with the International Prospective Register of Systematic Reviews for prevalence studies reporting CKD stages III-V from 2013 to 2021. The analysis sought to explain any significant differences in prevalence rates. The R statistical package was used for data analysis. Comparisons included measures of effect size due to the large sample sizes analysed. We also compared sex differences in prevalence rates, common aetiologies, and type of study methodologies employed.

**Results:**

Eight studies were analysed, with two from each region. The matched prevalence rates of CKD between the various regions and South Africa showed significant differences, except for one comparison between South Africa and an African study [*p* = 0.09 (95% CI − 0.04–0.01)]. Both sub-Saharan African studies had a higher prevalence than South Africa. One study in Africa had a higher prevalence, while the other had a lower prevalence, whilst one Global study had a higher prevalence, and the other had a lower prevalence compared to South Africa. The statistical differences analysed using the Cramer’s V test were substantially less than 0.1. Thus, differences in comparisons were largely due to differences in sample sizes rather than actual differences.

**Conclusion:**

Variable prevalence rates between regions included disparities in sample size, definitions of CKD, lack of chronicity testing and heterogeneous laboratory estimations of eGFR. Improved consistency and enhanced methods for diagnosing and comparing CKD prevalence are essential.

**Supplementary Information:**

The online version contains supplementary material available at 10.1186/s12882-023-03109-1.

## Background

The estimated number of people with chronic kidney disease (CKD) globally is approximately 844 million [1]. Patients with CKD are estimated to be twice the number of people with diabetes worldwide and more than twenty times the number of people affected by human immunodeficiency virus/acquired immune deficiency syndrome (HIV/AIDS) [1]. Kidney diseases are among the most common global non-communicable diseases (NCDs) [1]. The worldwide all-age prevalence of CKD has increased by 29.3% over the past three decades [2]. CKD has therefore become a universal public health priority [3]. Even though CKD prevalence has been researched more widely in economically developed countries, the disease burden is even more significant in developing countries [4]. The systematic review by Mills et al. estimated the global prevalence of CKD to be 11.1% [4]. However, the numbers affected by CKD rest on data of various qualities, approximations, and assumptions [1, 5]. It is acknowledged that CKD is common, but the challenge is to define its true prevalence [5].

Non-communicable diseases (NCDs) are increasingly contributing to morbidity and mortality over the last three decades [6]. The factors contributing to NCDs rise are increasing longevity, urbanization, and cultural changes [6]. Metabolic disorders such as diabetes mellitus have contributed heavily to NCD deaths [7]. There is a projected increase of 156% in diabetes mellitus, with about 25 million more cases estimated from 2017 to 2045 [7]. The high estimated prevalence of CKD will cause a significant disruption of healthcare provision obliging fundamental infrastructural changes with increasing expenditure [8]. Comparative CKD prevalence studies involving different countries or within a continent have revealed statistically significant differences in prevalence rates [9, 10]. The variances proposed were due to actual differences and disparities in study methods [9, 10].

Due to the dearth of epidemiological data from the majority of the continent, the prevalence of CKD in Africa continues to be underestimated [11] The majority of CKD prevalence studies conducted in Africa are not optimal [11, 12] Sub-Saharan Africa comprises 85% of the African population with a higher prevalence of CKD compared to the continent’s north [11]. The most frequent causes of CKD in Africa are hypertension and diabetes mellitus followed by chronic glomerulonephritis and tubulointerstitial disorders [11]. Poverty and a lower socioeconomic status are two independent risk factors for developing CKD in Africa and hasten the course of the disease [13].

The International Society of Nephrology (ISN) Global Health Atlas survey for Africa estimated the prevalence of CKD in South Africa to be 10.7% (95% CI 9.94–11.57) [14]. The distribution of NCDs in South Africa displays socioeconomic disparities, with the most onerous burden falling on poor communities in urban areas [15]. The World Health Organization (WHO) estimates that the burden of NCDs in South Africa is two to three times higher than in other developing countries [15].

The lack of comprehensive CKD registries in South Africa and the rest of Africa has resulted in limited knowledge of CKD prevalence. The ISN has underscored that the current and future burden of CKD will be concentrated in lower socioeconomic countries, which often lack systematized and coordinated policies to manage the problem [16]. Accurate CKD prevalence rates allow for efficient preparation and execution of intervention and prevention programs [17]. The purpose of this review is to compare the CKD prevalence rates in South Africa with prevalence rates in sub-Saharan Africa, Africa as a whole, and globally. The study sought to explain the causes of any substantial differences in prevalence rates if this was present.

## Method

The study was registered with the International Prospective Register of Systematic Reviews (PROSPERO). The reference number for the review was CRD42022330121. Two reviewers applied the eligibility criteria independently. Decisions were checked by a third reviewer. Disagreements were resolved through discussion and reaching a consensus. The study searched for publications on CKD using Google Scholar, Scopus, Embase, and PubMed/Medline.

The search terms included “prevalence,“ “epidemiology,” “chronic kidney disease,“ “renal insufficiency,” “renal impairment,” “nephropathy,” “stage III-V CKD,” “proteinuria, “albuminuria,” “meta-analysis,” “systematic reviews,” “cohort,” “cross-sectional,” “population-based,” “South Africa,“ “sub-Saharan Africa,“ “Africa,” “global.

The current Kidney Disease Improving Global Outcomes (KDIGO) staging criteria for CKD were included (stage III-V); hence the period for the studies was limited predominantly to the last decade (2013–2021) [18]. The search included only those reporting CKD (stage III-V) prevalence as not all studies included stages I-II CKD. Inclusion criteria included adult studies and English language articles. Studies that were translated into English were also included. The search included meta-analyses, systematic reviews, cohort, and cross-sectional studies. The studies were expected to use the prevailing definition of CKD. Criteria were also limited to those directly reporting studies of CKD in South Africa, sub-Saharan Africa, and globally.

**Fig. 1:**
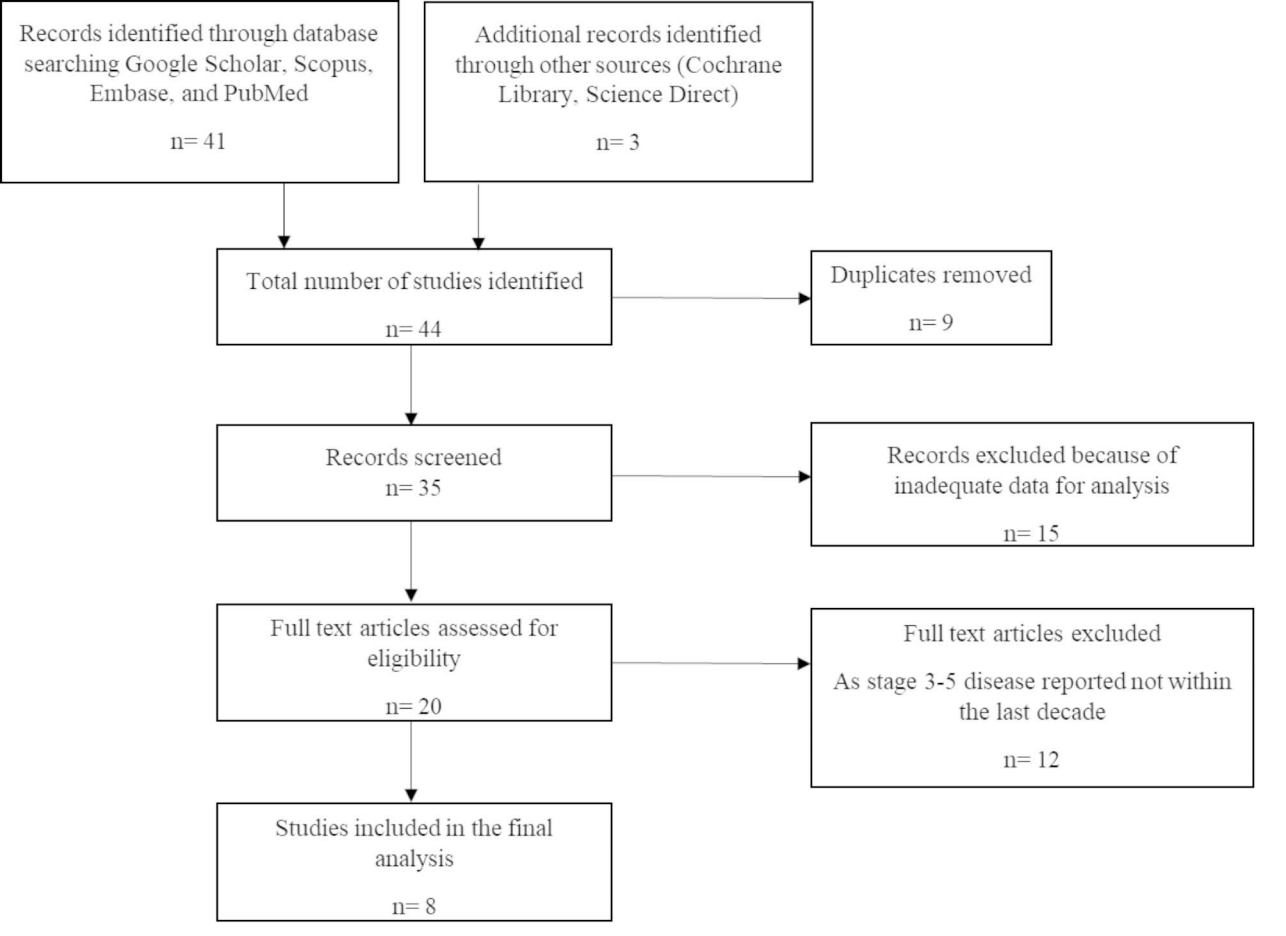
Selection of papers for analysis of the prevalence of CKD from South Africa, sub-Saharan Africa, Africa and Globally

Exclusion criteria were studies with patients under 12 years of age, those with inaccessible full texts, non-English studies that were not translated into English. Studies involving specific populations such as pregnant women, acute kidney injury or transplantation were excluded. (Fig. 1). The first reviewer developed a data extraction tool. The data extracted included author, year of study, region, the prevalence of CKD, study population, and study design. Information acquired was tabulated on an Excel Spreadsheet (Microsoft Office for Windows, version 10; Microsoft Corporation, Redmond, WA®) for analysis.

The prevalence of CKD in South African studies was compared with the prevalence in sub-Saharan Africa, Africa, and globally to determine if there were statistically significant differences. Some of the papers reviewed had studied large numbers of patients. It was hence necessary to use the effect size to assess the strength of correlations where the chi-square test of independence would have shown dependence.

The null hypothesis proposed that there was no statistically significant difference in the prevalence of CKD between South Africa and sub-Saharan Africa, Africa, and globally. The R package was used for data analysis. In R, the test for difference between two proportions and the chi-square test for independence provided the same chi-square and *p* values. Rejection of the null hypothesis could be interpreted as evidence that the variables being considered are statistically dependent. An alternative interpretation for the rejection was that the sample proportions being compared were significantly different.

The probability of finding a significant difference between proportions is increased with large sample sizes. The increased chi-square statistic may not represent a strong pattern of dependence between variables but reflects an increase in sample size. It was necessary to review the test of independence between two variables and use the effect size to assess whether significant differences were not due to large sample sizes. The Cramer’s V test was used as an effect size measurement for the chi-square test of independence. The test measured how strongly categorical fields, regions, and CKD are associated.

## Results

The analysis incorporated eight studies. (Table 1).

**Table 1:** Studies in South Africa, sub-Saharan Africa, Africa, and globally on prevalence of CKD

Author	Region	Year	Study type	95% Confidence interval	Reported CKD III-V prevalence rate	CKD number of patients	Non-CKD number of patients	Total number of patients
Matsha et al. [19]	South Africa	2013	Cohort Population based	5.0-8.5	8.7%	104	1 111	1202
Adeniyi et al. [20]	South Africa	2016	Cohort Population based	3.2–9.7	6.4%	31	458	489
George et al. [8]	Sub-Saharan Africa	2019	Cross sectional Population based	9.9–11.7	10.7%	868	7242	8110
Stanifer *et a*l [22]	Sub-Saharan Africa	2014	Systematic review Population based	12.2–15.7	13.9%	8939	55,368	64,307
Kaze et al. [12]	Africa	2018	Systematic review Population and hospital based	3.3–6.1	4.6%	4528	9 3904	98 432
Abd ElHafeez et al. [11]	Africa	2018	Systematic review Population based	9.8–10.5	10.1%	15 150	134 850	150 000
Hill et al. [4]	Global	2016	Systematic review Population based	9.2–12.2	10.6%	742 000	6 258 000	7 000 000
Bikbov et al. [2]	Global	2017	Systematic review Population based	3.5–4.3	4.1%	314 262 509	7 350 676 734	7 664 939 243

There were two studies each from South Africa, sub-Saharan Africa, Africa, and globally. A total of 7 665 961 783 participants were included: 315 034 128 (4.1%) having CKD stages III-V. The sample size of the studies ranged from 489 in a South African study [19, 20] to 7 664 939 243 in a global study [2]. The prevalence rates for CKD ranged from 6.4 to 8.7% in South Africa [19, 20], 10.7–13.9% in sub-Saharan Africa [21, 22], 4.6–10.1% in Africa [16, 17] and 4.1–10.6% globally [2, 4].

Matsha et al. published a regional cohort study on CKD in the Western Cape, South Africa, in one of the two South African studies [19]. The age-standardized prevalence using the Chronic Kidney Disease Epidemiology Collaboration equation (CKD-EPI) of CKD was 8.7% (95% CI 7.5–9.9) [19]. The overall mean age of participants was 52.9 ± 14.8 years; females constituted 75.3% of the study group. The risk factors involved included hypertension (33.0%) which also doubled the risk of developing CKD [19]. The prevalence of diabetes was 26.0%, with obesity being a significant risk factor for developing diabetes mellitus [19]. The prevalence of HIV was not reported.

The second South African study was a cross-sectional survey of CKD prevalence from the Western Cape by Adeniyi et al. [20]. The age-standardized prevalence using the CKD-EPI equation for CKD was 6.4% (95% CI 3.2–9.7%) [20]. Patients had a mean age of 46.3 ±8.5 years with the majority (70.3%) being female [20]. Risk factors included hypertension and diabetes, with a prevalence of 55.2% and 20.7%, respectively, while the prevalence of HIV was not reported [20].

In the sub-Saharan African study by George et al. in 2019, using a population-based study, the authors investigated the CKD prevalence in four sub-Saharan countries, viz. Burkina Faso, Ghana, Kenya, and South Africa [21]. The overall prevalence of CKD was 10.7% (95% CI 9.9–11.7) [10]. South Africa had the highest prevalence of 12.9% (95% CI 10.6–11.5) compared to the East and West African countries [21]. The mean age of participants was 49.9 ±5.8 years [10]. Females accounted for 49.2% of the study participants [21]. Women had a higher prevalence of CKD of 12.0% (95% CI 10.8–13.2) compared to men, with a prevalence of 9.5% (95% CI 8.3–10.8) [10]. Prevalence of the risk factors of hypertension, diabetes, and HIV were 32.6% (95% CI 31.3–34), 5.6% (95% CI 5-6.2), and 15.9% (95% CI 14.9–17.1), respectively [21].

A systematic review by Stanifer et al. in 2014 of 22 medium and high-quality studies in sub-Saharan Africa reported the prevalence of CKD to be 13.9% (95% CI 13.8–19.6) [22]. The mean age in the different quality studies was 41.5± 4.1 years, with females constituting 57.5% of participants [22]. Risk factors included hypertension and diabetes, and HIV, with a median prevalence of 16.8% and 17.1%, and 11.9%, respectively [22].

In a meta-analysis of 98 CKD studies in Africa by Kaze et al. in 2018, the overall prevalence of CKD was 4.6% (95% CI 3.3–6.1) [12]. The mean age of participants was 43.0 ± 6.2 years. [12] The proportion of female participants was not reported. The main risk factors for CKD were hypertension, diabetes, and HIV [12]. The prevalence rates for the risk factors were 35.6% (95% CI 27.9–43.7), 13.3% (95% CI 10.7–16), and 17.9% (95% CI 10.9–26.1), respectively [12].

In another systematic review of 152 CKD stage III-V prevalence studies in Africa in 2018 by Abd El Hafeez et al. [11], the CKD prevalence rate was 10.1% (95% CI 9.8–10.5) [11]. The median age was 52.8 ± 11.7 years [11]. The overall proportion of female participants was 64.3% [11]. The pooled risk factor prevalence of hypertension was 34.5% (95% CI 34.0–36.0), diabetes 24.7% (95 CI 23.6–25.7), and HIV 5.6% (95% CI 5.4–5.8) [11].

The global study by Hill et al. in 2016 was a systematic review and meta-analysis of 100 observational studies involving seven million patients [4]. The estimated prevalence of CKD was 10.6% (95% CI 9.2–12.2%) [4].The mean age of all participants was 49.0 ± 8.5 years [4]. The proportion of female participants studied was 55.0% [4]. The prevalence of CKD in males was 8.1% (95% CI 6.3–10.2) [4]. The CKD prevalence in females was 12.1% (95% CI 10.6–13.8) [4]. The median prevalence of the two major risk factors was hypertension (40.1%) and diabetes mellitus (15.1%) [4]. HIV was not reported as a risk factor.

Bikbov et al. in 2020 reported a systemic analysis of the Global Burden of Disease (GBD) study based on published literature, registration systems, chronic kidney failure registries, and household surveys [2]. The estimated prevalence in a study population for CKD stage III was 3.9% (95% CI 3.5–4.3%), 0.16% (95% CI 0.0.13–0.19%) for CKD stage IV, and 0.07% (95% CI 0.06–0.08%) for CKD stage V [2]. The mean age and proportion of female participants were not reported, but the prevalence of CKD in females was 1.29-fold (95% CI 1.28–1.3) more than in males [2]. The age-standardized prevalence of CKD in females was 9.5% (95% CI 8.8–10.2] and 7.3% (95%CI 6.8–7.9) in males [2]. Major risk factors for CKD in the study were hypertension, with a prevalence of 43.2% (95% CI 42.3–54.1), and diabetes, with a prevalence of 57.6% (95% CI 50.5–63.8) [2]. There was no reporting of HIV as a risk factor.

The first comparison was between South Africa and sub-Saharan Africa. Both sub-Saharan studies had a higher prevalence of CKD compared to Matsha et al. [19]. Once more, when compared to Adeniyi et al. [20], both sub-Saharan studies revealed a higher prevalence of CKD. .

When comparing South Africa with Africa, only one study comparing Adeniyi et al. [20](South Africa) versus Kaze et al. [12] (Africa) displayed no significant difference. The African study by Kaze et al. [12] displayed a lower CKD prevalence. Abd El Hafeez et al. [11](Africa) had a higher prevalence of CKD than both South African studies. (Table 2)

**Table 2:** shows the results of statistical tests of differences in CKD prevalence in South Africa versus sub-Saharan Africa, African and global studies

Comparative Region	Authors	Proportions	95% CI	Chi-Squared	*p*-value	Cramer’s V
Sub-Saharan Africa	Matsha et al. [19] (SA)	0.07570715	-0.08: − 0.05	39.249	< 0.001	0.025
	Stanifer et al. [22]	0.13900508				
	Matsha et al. [19] (SA)	0.07570715	-0.05 : -0.01	10.78	< 0.001	0.035
	George et al. [8]	0.10702836				
	Adeniyi et al. [20] (SA)	0.06339468	-0.10 : -0.05	22.633	< 0.001	0.019
	Stanifer et al. [22]	0.13900508				
	Adeniyi et al. [20] (SA)	0.06339468	-0.07 : -0.02	8.919	< 0.001	0.033
	George et al. [8]	0.10702836				
Africa	Matsha et al. [19] (SA)	0.07570715	0.01 : 0.04	23.035	< 0.001	0.015
	Kaze et al. [12]	0.04600130				
	Matsha et al. [19] (SA)	0.07570715	-0.04 : -0.01	8.1396	0.004	0.007
	Abd ElHafeez et al. [11]	0.10100000				
	Adeniyi et al. [20] (SA)	0.06339468	-0.01 : 0.04	2.9644	0.090	0.006
	Kaze et al. [12]	0.04600130				
	Adeniyi et al. [20] (SA)	0.06339468	-0.06 : -0.01	7.1905	0.007	0.007
	Abd ElHafeez et al. [11]	0.10100000				
Global	Matsha et al. [19] (SA)	0.07570715	-0.04 : -0.01	11.321	< 0.001	0.001
	Hill et al. [4]	0.106				
	Adeniyi et al. [20](SA)	0.06339468	-0.06 : -0.02	8.9222	< 0.001	0.001
	Hill et al. [4]	0.106				
	Matsha et al. [19](SA)	0.07570715	0.02 : 0.05	35.947	< 0.001	0.00
	Bikbov et al. [2]	0.0410000				
	Adeniyi et al. [20] (SA)	0.06339468	-0.0002 : 0.04	5.6807	0.017	0.00
	Bikbov et al. [2]	0.0410000				

The final CKD prevalence comparison was between South Africa and global studies. The global study by Bikbov et al. [2] had a lower prevalence of CKD, while the global study by Hill et al. [4] had a higher prevalence of CKD when both were compared to the South African studies.

Overall, there were statistically significant differences in comparisons between all studies, except for one study comparing South Africa against Africa. The prevalence of CKD in both sub-Saharan studies was higher than in South African studies. One African study had a lower prevalence of CKD than the South African studies, while the other had a higher prevalence. Similarly, one global study had a lower prevalence of CKD than the South African studies, while the global study had a higher prevalence. However, the maximum Cramer’s V value for all comparisons was 0.035, all considerably less than 0.1, which suggested that these statistical differences were an effect of sample size rather than actual differences.

Table 3 compares the studies analysed in each geographical region, incorporating the mean age range of participants, number of female participants, and prevalence of risk factors. In addition, the table compared whether the Kidney Disease Improving Global Outcomes (KDIGO) guidelines were used to define CKD, including whether testing for chronicity of more than three months duration was used for the diagnosis of CKD. Further comparisons included the type of serum creatinine assay used, estimating equations to calculate the estimated glomerular filtration rate (eGFR), and if ethnicity co-efficient were employed.

**Table 3:** Comparison of eGFR equations, the mean age of participants, number of female participants, and prevalence of risk factors and laboratory methods

Author	Region	Mean age of participants (Years)	Female Participants (Percentage)	Prevalence of Hypertension	Prevalence of Diabetes	Prevalence of HIV	KDIGO CKD criteria	Test for chronicity > 3 months	Serum creatinine measurement: enzyme or Jaffe	eGFR equations studied	Ethnicity co-efficient used
Matsha et al. [19]	South Africa	52.9 ± 14.8	75.3	33.0	26.0	Not mentioned	No	No	Enzyme	CKD-EPI MDRD Cockcroft-Gault	Measured with and without ethnic co-efficient
Adeniyi et al. [20]	South Africa	46.3 ±8.5	70.3	55.2	20.7	Not mentioned	No	No	Enzyme	CKD-EPI MDRD	No
George et al. [8]	Sub-Saharan Africa	49.9 ±5.8	49.2	32.6	5.6	15.9	Yes	No	Enzyme	CKD-EPI MDRD	Measured with and without ethnic co-efficient
Stanifer et al. [22]	Sub-Saharan Africa	41.5 ± 4.1	57.5	16.8	17.1	11.9	No	No	Not mentioned	MDRD Cockcroft-Gault	No
Kaze et al. [12]	Africa	43.0 ± 6.2	Not mentioned	35.6	13.3	17.9	No	No	Not mentioned	CKD-EPI MDRD Cockcroft-Gault Cystatin C	No
Abd ElHafeez et al. [11]	Africa	52.8 ±11.7	64.3	34.4	24.7	5.6	No	No	Enzyme and Jaffe	CKD-EPI MDRD Cockcroft-Gault	No
Hill et al. [4]	Global	49.0 ± 8.5	55.0	40.1	15.1	Not mentioned	No	No	Enzyme and Jaffe	CKD-EPI MDRD	No
Bikbov et al. [2]	Global	Not studied	Not mentioned	43.2	57.6	Not mentioned	Modelled	No	Not mentioned	CKD-EPI MDRD	No

Most study participants in all studies assessed were in the fourth to fifth decade of life. There was a predominance of female participants in the prevalence studies. Hypertension and diabetes mellitus were the most common risk factors in all studies, with HIV identified as a common risk factor in sub-Saharan Africa and Africa.

Only George et al. [21] (sub-Saharan African study) used the KDIGO definition of CKD. None of the selected studies considered chronicity of more than three months for CKD. Matsha et al. [19], Adeniyi et al. [20] (South Africa), and George et al. [21] (sub-Saharan Africa) calculated serum creatinine with the enzyme-linked assay. Abd ElHafeez et al. [11] (Africa) and Hill et al. [4] (Global) analysed serum creatinine that was calculated using the enzyme-linked and Jaffe assays. The CKD-EPI, Modification of Diet in Renal Disease **(**MDRD), and Cockcroft-Gault equations were the most widely used for the estimated glomerular filtration rate (eGFR). Matsha et al. [19] (South Africa) and George et al. [21] (sub-Saharan Africa) calculated the eGFR with and without ethnicity co-efficient.

## Discussion

On *prima facie* analysis, there were statistically significant differences in CKD prevalence rates between South Africa and sub-Saharan Africa, Africa, and globally in all except for one comparison. The single comparison that did not show a statistically significant difference in CKD prevalence was between the South African study by Adeniyi et al. [20] compared to the African study by Kaze et al. [12]. The prevalence of CKD in sub-Saharan studies was higher than those in South African studies. However, it could not be determined whether the prevalence of CKD in South Africa was higher or lower than the African and global prevalence.

The wide variations in sample sizes between comparative groups limited the interpretation of statistical tests such as the *p*-values and confidence intervals [23]. The significant differences in prevalence may be due to large sample sizes. The analysis of the Cramer’s V effect size indicated a weak association between CKD prevalence rates and the regions. The statistically significant differences in prevalence rates across the regions may be due to differences in sample sizes rather than dependence of CKD prevalence between each geographical region. Our analysis shows a similarity to previous comparative studies between geographical regions [9, 10, 15]. Differences in prevalence rates of CKD between countries and regions have been documented, with variations being due to true differences or limitations caused by the heterogeneity of studies [15]. True variations result from high protein diets, smoking, physical activity, socioeconomic status, ethnicity, genetics, and birth weight [15]. International comparisons of CKD prevalence have been hindered by differences in age, sex distribution, sampling, and definitions of CKD [15]. Regional variations of CKD prevalence within a country are also frequent, and the degree of variations may fluctuate [15]. A rapid epidemiological transition could also explain the different prevalence of environmental changes, adoption of western lifestyles, and rapid urbanisation in Africa [12]. The clinical, demographic, and laboratory causes of variations in CKD prevalence will be discussed.

The median age of developing CKD in lower-middle-income countries was 43.7 years [12]. Observational and cohort studies in Africa have consistently shown an increased risk of cardiovascular disease mortality in the early stages, with nearly 40% of deaths from CKD occurring before 65 years [12]. The mean age of patients diagnosed with CKD in South Africa, sub-Saharan Africa, and Africa was younger, between the fourth and fifth decade, compared to the global CKD study by Hill et al. [4], where the highest prevalence was between the fifth and sixth decade. Lower kidney function was associated with a significant and progressive reduction of life expectancy in middle age for both men and women [24]. An earlier age at diagnosis heralds a worse prognosis.

The KDIGO criteria, if used to define stages of CKD, result in a considerable increase in prevalence with age and the method used to estimate GFR [25]. The threshold of 60ml/min/1.73m^2^ for the diagnosis of CKD could contribute to an overestimation of CKD in older patients [26]. Elderly populations exhibit a normal “physiological” decline in GFR with aging (renal senescence) [27]. Epidemiologic studies using a “once-off” testing of eGFR, especially with elderly participants, may also overestimate the burden of CKD in older patients [25]. The controversy of whether the decline in GFR is due to aging, as opposed to disease, has not been directly resolved [25]. A suggested method to overcome false positives would be to use the third percentile of eGFR creatinine levels and age-calibrated thresholds [26]. Alternatively, the Berlin Initiative Study 1 equation would be more suitable for subjects older than 70 years [28].

Most patients diagnosed with CKD were female, in keeping with the majority of worldwide CKD prevalence studies [29]. The prevalence of CKD in the United States of America is higher in females than males, but males have a higher prevalence of newly treated chronic kidney failure (CKF) [30]. The cause of this was indeterminate but may be multifactorial [3]. There is a possibility of overdiagnosis of CKD in older women than in men [31]. Women, on average, have lower estimated GFR and measured GFR (uncorrected for body surface area) and tend to progress to a GFR value of < 60ml/min/1.73m^2^ before men, although men progress more rapidly to CKF [31]. This physiological sex difference could contribute to an overdiagnosis of CKD in women than men as they age, especially in the absence of albuminuria [31].

The role of the social environment and economic conditions is an emerging component in the pathway from CKD risk to the development and complications of CKD and chronic kidney failure [32]. Socioeconomically underprivileged inhabitants worldwide show an unevenly high burden of CKD [32]. The burden is compounded by the inability to receive evidence-based care leading to poor clinical outcomes [32]. Lower socioeconomic status was related to a greater risk of prevalence of CKD [32].The poor with a higher kidney disease burden often have fewer resources to meet treatment costs [32]. The consequence is “catastrophic spending” (defined as out-of-pocket payments above 40% of non-food expenditure) [32]. Thus, advanced CKD could be considered a risk factor for poverty along with low education level, employment status, and ethnicity [33]. The entire family becomes affected by the reduction in resources [33]. Poverty can also directly affect adherence to medical treatment as the affected patient may be unable to access follow-up care or afford kidney replacement therapy when required [33].

Countries with a higher CKD prevalence have a higher risk factor profile [10]. Sub-Saharan Africa is estimated to have 18.65 million people with diabetes mellitus [22]. A similar number is estimated to develop hypertension by the end of this decade [22]. There would also be an estimated 22 million people living with HIV/AIDS during this time, posing a further substantial burden of CKD in this region [22]. In Africa, the dominant risk factors for developing CKD are hypertension, diabetes mellitus, and HIV [11, 12]. Africa also has the highest prevalence of HIV-1 infection [34]. There is a robust association between Apolipoprotein L1 gene variants found only on African chromosomes resulting in an increased probability of developing focal segmental glomerulosclerosis and HIV-associated nephropathy [34, 35]. Resource limitations lead to the late initiation of ARVs (antiretroviral agents), which predisposes to HIV-associated nephropathy [36]. The combination of genetic susceptibility with delayed treatment of HIV contributes to the increase in CKD prevalence and disease burden. Africa is, therefore, subject to a dual burden of non-communicable and endemic infectious diseases such as HIV leading to CKD [37].

Global studies identified hypertension, diabetes mellitus, female sex, and increasing age as the major risk factors for the development of CKD [2, 4]. International differences in the prevalence of risk factors for CKD could be affected by sample selection [10]. CKD prevalence fluctuates with time, as some international differences in CKD prevalence may be explained by differences in the study periods and the associated transition of risk factor profiles [10]. Increased prevalence within some regions compared to neighbouring areas with similar demographics may also indicate increased recognition and recording of CKD [29]. The epidemiological transition from communicable to non-communicable diseases, with significant increases in hypertension and diabetes mellitus with aging, may also account for the increased prevalence of CKD [11, 38, 39].

The estimated requirements for kidney replacement therapy will double from 2.62 million in 2010 to 5.4 million people by 2030 [40]. Global deaths due to kidney disease are projected at between 5 and 10 million people annually due to a shortage of kidney replacement therapy services [40]. Higher-income countries spend 2–3% of their annual health budget on CKF treatment for approximately 0.03% of the total population [40]. Lower-income countries are not able to provide similar resources for chronic kidney failure (CKF). They will most likely experience the societal, health, and economic burden of mostly untreated CKF.

Over the past decade, there have been substantial developments in standardising assays for serum creatinine [41]. The re-calibration of serum creatinine assays to an isotope dilution mass spectrometry reference method has resulted in more specific assays traceable to the International System of units [42]. The introduction of isotope dilution mass spectrometry calibration for serum creatinine assays has addressed the variability of serum creatinine data [42]. However, difficulties persist concerning using eGFR to assess CKD prevalence in epidemiological studies [42]. A continuing complication is that the effect of assay calibration differs between eGFR equations [43]. Variations in calibration have a more significant effect on the MDRD equation than on the CKD-EPI equation for eGFR [43]. The variation is due to the mathematical exponent applied to serum creatinine in elevated eGFR ranges and is lower in CKD-EPI than the MDRD equation [43]. The CKD-EPI equation gives a lower prevalence of CKD due to a higher eGFR in general or specific population participants than other equations [43]. In contrast, the systematic underestimation of eGFR with the MDRD equation is associated with an overestimation of CKD prevalence in epidemiological studies [43].

The lack of standardized equations to calculate eGFR was highlighted in the studies by several authors in this paper [2, 4, 17, 20–22]. Most studies reviewed displayed an analytical heterogeneity used to measure creatinine. Evaluation of eGFR is fundamental to medical practice, research, and public health [44]. Serum creatinine is the most commonly utilized biomarker to assess eGFR [45]. However, individual values may vary due to factors that include mass, age, sex, ethnicity, and diet unrelated to CKD [45]. Measured GFR (mGFR) and gold-standard measurements using inulin clearance are, unfortunately, too cumbersome to perform in extensive epidemiologic studies [31]. In a collaborative study from Malawi, Uganda, and South Africa that prospectively measured kidney function, it was established that creatinine-based GFR-estimating equations overestimate kidney function [46]. The implication is that the burden of kidney disease may be significantly underestimated in Africa [46].

A common limitation in CKD prevalence studies is the “once-off testing” of serum creatinine (and hence eGFR). Other limitations included quantifying albuminuria; the different formulae used to calculate eGFR, the absence of proteinuria and haematuria testing, and heterogeneity in sample data used to calculate the prevalence of CKD. Once off, eGFR testing or confirming chronicity was reported here as a limitation in numerous studies [2, 4, 7, 8, 16, 20]. Glassock et al. contend that although CKD is widespread, the contention that the prevalence is increasing in many countries may be incorrect [31]. The authors maintain that using “once-off testing” of eGFR and albuminuria to define prevalence in epidemiological studies is controversial, as these “single test” studies do not adhere to the KDIGO CKD definition of three-month duration [18]. The “once-off” testing produces a false positive diagnostic rate of about 30% for eGFR and even higher for albuminuria [47]. Conversely, false-negative results, which primarily involve the younger population, arise when they have an eGFR above 60 ml/min/1.73m^2^ [48]. This subset does not meet the criteria for the definition of CKD and is without proteinuria, but they have a low eGFR for their age, below the 3rd percentile for age and sex category [48].

Using ancestry coefficients, sex, and age of patients can further contribute to the limitations of prevalence studies. The ancestry coefficient is a significant constituent of the MDRD and CKD-EPI equations [31]. It was recommended to improve the understanding of the prevalence of CKD in ethnically diverse populations [31]. However, the African American coefficient results in the MDRD and CKD-EPI equations for eGFR being 21% and 15% more elevated, respectively, than the same equations without coefficients [31]. It can be contended that the use of race in eGFR equations is a social and not a biological concept [46]. The inclusion of race ignores diversity within and among racial groups [46]. Alterations in estimating equations can affect the calculation of the burden of CKD and potentially disrupt patient care [46]. It can also be debated that keeping a race term in GFR equations adversely affects access to kidney replacement therapy [49].

Alternatives to calculating eGFR without using race are currently being evaluated [50]. The estimation of GFR with the usage of cystatin C was similar to estimations using serum creatinine [50]. Cystatin C-based estimations did not use race or ancestry and were not enhanced or changed by their inclusion [50]. Most recent eGFR equations use creatinine and cystatin C without race [51]. They are more accurate in estimating GFR than either equation using creatinine or cystatin C alone [51]. This has resulted in reduced differences from measured GFR between race groups [51]. A systematic review of epidemiological studies from sub-Saharan Africa highlighted the source’s potential for bias [52]. These include variability in the requirements for serum creatinine assays, appropriate choice of estimating equations to calculate eGFR, and appropriate diagnostic criteria for CKD [52]. The results were consistent with other worldwide studies [52]. The ongoing evolution of data from eGFR equations will further inform clinical practice, research, and public health considerations [52].

An essential requirement for the management of CKD is for efficient and sustainable solutions to capture high-quality population-based health data and extrapolate it into health information systems [53]. This will allow a better understanding of CKD epidemiology and variations in CKD prevalence [53]. The CKD in Africa (CKD-Africa) project is a continental collaboration network that aims to provide uniformly reliable estimates for CKD prevalence [53]. The collaboration has currently networked 12 African countries in sub-Saharan Africa, totalling 39 studies and 35 747 participants [53]. This collective health system would be able to effectively advise future health services planning and policy for CKD management in Africa [53].

The study limitations include analysing two studies from South Africa from the same region. These studies may not represent the country’s prevalence of CKD because regional variations in CKD prevalence can occur within a country [15]. The South African studies had relatively small numbers of participants compared to those in sub-Saharan Africa, Africa, and globally. HIV, a significant risk factor for CKD in sub-Saharan Africa, was not investigated amongst participants in the South African studies. The population sampling was also not representative of the South African population demographics. A further limitation was the low number of studies that were eligible for inclusion in the analysis.

## Conclusion

There was a statistically significant variation in the prevalence of CKD between South Africa and sub-Saharan Africa, Africa, and globally in all except one comparison. However, there was a poor correlation due to the effect size, which suggests that these differences may be due to comparing studies with large sample sizes than to actual differences in the prevalence. This review echoed the marked heterogeneity when comparing CKD prevalence from different regions. These included varying sample sizes, differences in the study methodology, the criteria for the definition of CKD, the lack of chronicity reporting, and variances in serum creatinine measurements leading to variable eGFRs. Enhanced uniformity and novel approaches are crucial for performing and reporting CKD prevalence studies to advance the accuracy of comparing the burden of the disease.

## Electronic supplementary material

Below is the link to the electronic supplementary material.

Supplementary Table 1: Search Strategy

## Data Availability

Datasets generated and/or analysed during the current study are available upon request. Kindly contact the corresponding author of the study, Dr.SP Hariparshad at Sudeshph@yahoo.com.

## List of Abbreviations

ARV: antiretroviral
CKD: Chronic kidney disease
CKD-EPI: Chronic Kidney Disease Epidemiology Collaboration
CKF: chronic kidney failure
eGFR: estimated glomerular filtration rate
GBD: Global Burden of Disease study
HIV/AIDS: Human immunodeficiency virus/acquired immune deficiency syndrome
ISN: International Society of Nephrology
KDIGO: Kidney Disease Improving Global Outcomes
MDRD: Modification of Diet in Renal Disease
mGFR: measured glomerular filtation rate
NCD: non-communicable disease
PROSPERO: International Prospective Register of Systematic Reviews
WHO: World Health Organisation

## References

[1] Jager KJ, Kovesdy C, Langham R, Rosenberg M, Jha V, Zoccali C. A single number for advocacy and communication-worldwide more than 850 million individuals have kidney diseases. Oxford University Press; 2019. pp. 1803–5. 10.1093/ndt/gfz174 10.1093/ndt/gfz174 31566230

[2] Bikbov B, Purcell CA, Levey AS, Smith M, Abdoli A, Abebe M (2020). Global, regional, and national burden of chronic kidney disease, 1990–2017: a systematic analysis for the global burden of Disease Study 2017. The Lancet.

[3] Levin A, Tonelli M, Bonventre J, Coresh J, Donner J-A, Fogo AB (2017). Global kidney health 2017 and beyond: a roadmap for closing gaps in care, research, and policy. The Lancet.

[4] Hill NR, Fatoba ST, Oke JL, Hirst JA, O’Callaghan CA, Lasserson DS (2016). Global prevalence of chronic kidney disease–a systematic review and meta-analysis. PLoS ONE.

[5] Black C, van der Veer SN. Unlocking the value of variation in CKD prevalence. Am Soc Nephrol. 2016;1874–7. 10.1681/asn.2015111280 10.1681/ASN.2015111280 PMC4926991 26714870

[6] Matsha TE, Erasmus RT (2019). Chronic kidney disease in sub-saharan Africa. The Lancet Global Health.

[7] Saeedi P, Petersohn I, Salpea P, Malanda B, Karuranga S, Unwin N (2019).

[8] George JA, Brandenburg J-T, Fabian J, Crowther NJ, Agongo G, Alberts M (2019). Kidney damage and associated risk factors in rural and urban sub-saharan Africa (AWI-Gen): a cross-sectional population study. The Lancet Global Health.

[9] Wang F, He K, Wang J, Zhao M-H, Li Y, Zhang L (2018). Prevalence and risk factors for CKD: a comparison between the adult populations in China and the United States. Kidney Int Rep.

[10] Stel VS, Brück K, Fraser S, Zoccali C, Massy ZA, Jager KJ (2017). International differences in chronic kidney disease prevalence: a key public health and epidemiologic research issue. Nephrol Dialysis Transplantation.

[11] Abd ElHafeez S, Bolignano D, D’Arrigo G, Dounousi E, Tripepi G, Zoccali C (2018). Prevalence and burden of chronic kidney disease among the general population and high-risk groups in Africa: a systematic review. BMJ open.

[12] Kaze AD, Ilori T, Jaar BG, Echouffo-Tcheugui JB (2018). Burden of chronic kidney disease on the african continent: a systematic review and meta-analysis. BMC Nephrol.

[13] Nyirenda MJ (2016). Non-communicable diseases in sub-saharan Africa: understanding the drivers of the epidemic to inform intervention strategies. Int health.

[14] Bello AK, Levin A, Lunney M, Osman MA, Ye F, Ashuntantang G, Bellorin-Font E, Benghanem GM, Ghnaimat M, Harden P (2019). Global kidney Health Atlas: a report by the International Society of Nephrology on the global burden of end-stage kidney disease and capacity for kidney replacement therapy and conservative care across world countries and regions.

[15] Mayosi BM, Flisher AJ, Lalloo UG, Sitas F, Tollman SM, Bradshaw D (2009). The burden of non-communicable diseases in South Africa. The lancet.

[16] Working Group International Society of Nephrology ISN Framework for Developing (2021). Dialysis Programs in low-resource settings.

[17] Brück K, Stel V, Gambaro G, Hallan S, Völzke H, Ärnlöv J (2015). European CKD Burden Consortium: CKD prevalence varies across the european general population. J Am Soc Nephrol.

[18] Improving Global Outcomes (KDIGO) CKD Work Group (2013). KDIGO 2012 clinical practice guideline for the evaluation and management of chronic kidney disease. Kidney Int Suppl.

[19] Matsha TE, Yako YY, Rensburg MA, Hassan MS, Kengne AP, Erasmus RT (2013). Chronic kidney diseases in mixed ancestry south african populations: prevalence, determinants and concordance between kidney function estimators. BMC Nephrol.

[20] Adeniyi AB, Laurence CE, Volmink JA, Davids MR (2017). Prevalence of chronic kidney disease and association with cardiovascular risk factors among teachers in Cape Town, South Africa. Clin kidney J.

[21] George JA, Brandenburg JT, Fabian J, Crowther NJ, Agongo G, Alberts M, Ali S, Asiki G, Boua PR, Gómez-Olivé FX, Mashinya F. Kidney damage and associated risk factors in rural and urban sub-Saharan Africa (AWI-Gen): a cross-sectional population study. The Lancet Global Health. 2019 Dec1; 7(12):e1632-4310.1016/s2214-109x(19)30443-7 10.1016/S2214-109X(19)30443-7 PMC7033368 31708144

[22] Stanifer JW, Jing B, Tolan S, Helmke N, Mukerjee R, Naicker S (2014). The epidemiology of chronic kidney disease in sub-saharan Africa: a systematic review and meta-analysis. The Lancet Global Health.

[23] Shreffler J, Huecker MR. Hypothesis testing, P values, confidence intervals, and significance. StatPearls [Internet] Treasure Island (FL): StatPearls Publishing; 2022 Jan–. PMID: 32491353. 32491353

[24] Turin TC, Tonelli M, Manns BJ, Ravani P, Ahmed SB, Hemmelgarn BR (2012). Chronic kidney disease and life expectancy. Nephrol Dialysis Transplantation.

[25] Glassock R, Delanaye P, El Nahas M (2015). An age-calibrated classification of chronic kidney disease. JAMA.

[26] Delanaye P, Glassock RJ, De Broe ME (2017). Epidemiology of chronic kidney disease: think (at least) twice!. Clin Kidney J.

[27] Glassock RJ, Winearls C (2008). The global burden of chronic kidney disease: how valid are the estimates?. Nephron Clin Pract.

[28] Matašin M, Domislović V, Fuček M, Gellineo L, Jelaković A, Dika Ž (2021). Berlin Initiative Study 1 equation and HUGE formula for more accurate estimation of kidney function in elderly. Rad Hrvatske akademije znanosti i umjetnosti Medicinske znanosti.

[29] Carrero JJ, Hecking M, Chesnaye NC, Jager KJ (2018). Sex and gender disparities in the epidemiology and outcomes of chronic kidney disease. Nat Rev Nephrol.

[30] Coresh J, Selvin E, Stevens LA, Manzi J, Kusek JW, Eggers P (2007). Prevalence of chronic kidney disease in the United States. JAMA.

[31] Glassock RJ, Warnock DG, Delanaye P (2017). The global burden of chronic kidney disease: estimates, variability and pitfalls. Nat Rev Nephrol.

[32] Nicholas SB, Kalantar-Zadeh K, Norris KC. Socioeconomic disparities in chronic kidney disease. Advances in chronic kidney disease. 2015;22(1):6–15. 10.1053/j.ackd.2014.07.002 10.1053/j.ackd.2014.07.002 PMC4291541 25573507

[33] Morton RL, Schlackow I, Gray A, Emberson J, Herrington W, Staplin N (2018). Impact of CKD on household income. Kidney Int Rep.

[34] Kasembeli AN, Duarte R, Ramsay M, Mosiane P, Dickens C, Dix-Peek T (2015). APOL1 risk variants are strongly associated with HIV-associated nephropathy in black South Africans. J Am Soc Nephrol.

[35] Kopp JB, Nelson GW, Sampath K, Johnson RC, Genovese G, An P (2011). APOL1 genetic variants in focal segmental glomerulosclerosis and HIV-associated nephropathy. J Am Soc Nephrol.

[36] Szczech LA. Renal disease: the effects of HIV and antiretroviral therapy and the implications for early antiretroviral therapy initiation. Current Opinion in HIV and AIDS. 10.1097/coh.0b013e328329c6462009;4(3):167-70. 10.1097/COH.0b013e328329c646 19532045

[37] Hodel NC, Hamad A, Praehauser C, Mwangoka G, Kasella IM, Reither K (2018). The epidemiology of chronic kidney disease and the association with non-communicable and communicable disorders in a population of sub-saharan Africa. PLoS ONE.

[38] Cockwell P, Fisher L-A (2020). The global burden of chronic kidney disease. The Lancet.

[39] Madala ND, Thusi GP, Assounga AG, Naicker S (2014). Characteristics of south african patients presenting with kidney disease in rural KwaZulu-Natal: a cross sectional study. BMC Nephrol.

[40] Luyckx VA, Tonelli M, Stanifer JW (2018). The global burden of kidney disease and sustainable development goals. Bull World Health Organ.

[41] Piéroni L, Delanaye P, Boutten A, Bargnoux A-S, Rozet E, Delatour V (2011). A multicentric evaluation of IDMS-traceable creatinine enzymatic assays. Clin Chim Acta.

[42] Delanaye P, Cavalier E, Cristol J-P, Delanghe JR (2014). Calibration and precision of serum creatinine and plasma cystatin C measurement: impact on the estimation of glomerular filtration rate. J Nephrol.

[43] White SL, Polkinghorne KR, Atkins RC, Chadban SJ (2010). Comparison of the prevalence and mortality risk of CKD in Australia using the CKD epidemiology collaboration (CKD-EPI) and modification of Diet in Renal Disease (MDRD) Study GFR estimating equations: the AusDiab (australian diabetes, obesity and lifestyle) study. Am J Kidney Dis.

[44] Levey AS, Coresh J, Tighiouart H, Greene T, Inker LA (2019). Strengths and limitations of estimated and measured GFR. Nat Rev Nephrol.

[45] Centres for disease control and Prevention. Chronic kidney disease in the United States., 2019. Atlanta, GA: US Department of Health and Human Services, Centers for Disease Control and Prevention. 2019. March,3

[46] Fabian J, Kalyesubula R, Mkandawire J, Hansen CH, Nitsch D, Musenge E (2022). Measurement of kidney function in Malawi, South Africa, and Uganda: a multicentre cohort study. The Lancet Global Health.

[47] Eriksen B, Ingebretsen O (2006). The progression of chronic kidney disease: a 10-year population-based study of the effects of gender and age. Kidney Int.

[48] Gharbi MB, Elseviers M, Zamd M, Alaoui AB, Benahadi N, Trabelssi EH (2016). Chronic kidney disease, hypertension, diabetes, and obesity in the adult population of Morocco: how to avoid “over”-and “under”-diagnosis of CKD. Kidney Int.

[49] Delgado C, Baweja M, Burrows NR, Crews DC, Eneanya ND, Gadegbeku CA et al. Reassessing the inclusion of race in diagnosing kidney diseases: An interim report from the NKF-ASN task force. Am J Kidney Dis. 2021 Jul; 78(1):103–115. 10.1053/j.ajkd.2021.03.008 10.1053/j.ajkd.2021.03.008 PMC8238889 33845065

[50] Inker LA, Eneanya ND, Coresh J, Tighiouart H, Wang D, Sang Y (2021). New creatinine-and cystatin C–based equations to estimate GFR without race. N Engl J Med.

[51] Hsu C-y, Yang W, Parikh RV, Anderson AH, Chen TK, Cohen DL (2021). Race, genetic ancestry, and estimating kidney function in CKD. N Engl J Med.

[52] Fabian J, George JA, Etheredge HR, van Deventer M, Kalyesubula R, Wade AN (2019). Methods and reporting of kidney function: a systematic review of studies from sub-saharan Africa. Clin kidney J.

[53] George C, Stoker S, Okpechi I, Woodward M, Kengne A. The Chronic Kidney Disease in Africa (CKD-Africa) collaboration: lessons from a new pan-African network. BMJ Global Health. 2021 Aug1; 6(8):e006454. 10.1136/bmjgh-2021-006454 10.1136/bmjgh-2021-006454 PMC8340290 34348933

