# Supplementary material for: The prevalence of chronic kidney disease in South Africa - limitations of studies comparing prevalence with sub-Saharan Africa, Africa, and globally
Source: BMC Nephrol. 2023 Mar 21;24:62. doi: 10.1186/s12882-023-03109-1 (PMC10029276; doi:10.1186/s12882-023-03109-1)
Supplement: Supplementary file 1 — Supplementary Table 1: Search Strategy [file 12882_2023_3109_MOESM1_ESM.docx]

Supplementary table 1: Search strategy

| Region | Search filter | Search terms used | Number of studies | Reason for exclusions |
| --- | --- | --- | --- | --- |
| South Africa | Google Scholar, Scopus, Embase , PubMed  Cochrane library, Science Direct | South Africa, prevalence, epidemiology, chronic kidney disease, renal insufficiency, renal impairment, nephropathy, stage III-V CKD, proteinuria, albuminuria, meta-analysis, systematic reviews, cohort, cross sectional, population-based | 8 | Inadequate data for analysis  Not reported in the last decade |
| sub-Saharan Africa | Google Scholar , Scopus, Embase , PubMed  Cochrane library, Science Direct | Sub-Saharan Africa, prevalence, epidemiology, chronic kidney disease, renal insufficiency, renal impairment, nephropathy, stage III-V CKD proteinuria, albuminuria, meta-analysis, systematic reviews, cohort, cross sectional, population-based | 6 | Inadequate data for analysis  Not reported in the last decade |
| Africa | Google Scholar , Scopus , Embase , PubMed  Cochrane library,  Science Direct | Africa, prevalence, epidemiology, chronic kidney disease, renal insufficiency, renal impairment, nephropathy, stage III-V CKD , proteinuria ,albuminuria, meta-analysis, systematic reviews , cohort , cross sectional, population-based | 12 | Inadequate data for analysis  Not reported in the last decade |
| Global | Google Scholar , Scopus, Embase , PubMed  Cochrane library , Science Direct | global, prevalence, epidemiology, chronic kidney disease, renal insufficiency, renal impairment, nephropathy, stage III-V CKD, proteinuria, albuminuria, meta-analysis, systematic reviews , cohort , cross sectional, population based | 9 | Inadequate data for analysis  Not reported in the last decade |
